# Apolipoprotein E Plays a Key Role against Cryptosporidial Infection in Transgenic Undernourished Mice

**DOI:** 10.1371/journal.pone.0089562

**Published:** 2014-02-28

**Authors:** Orleâncio G. R. Azevedo, David T. Bolick, James K. Roche, Relana F. Pinkerton, Aldo A. M. Lima, Michael P. Vitek, Cirle A. Warren, Reinaldo B. Oriá, Richard L. Guerrant

**Affiliations:** 1 Division of Infectious Diseases and International Medicine, Center for Global Health, School of Medicine, University of Virginia, Charlottesville, Virginia, United States of America; 2 Laboratory of Infectious Diseases, Clinical Research Unit, Institute of the Brazilian Semi-Arid, School of Medicine, Federal University of Ceara, Fortaleza, Ceará, Brazil; 3 Laboratory of the Biology of Tissue Healing, Ontogeny and Nutrition, Institute of the Brazilian Semi-arid, School of Medicine, Federal University of Ceara, Fortaleza, Ceará, Brazil; 4 Duke University Medical Center, Department of Medicine, Durham, North Carolina, United States of America; Indian Institute of Science, India

## Abstract

Apolipoliprotein E (apoE), a critical targeting protein in lipid homeostasis, has been found to have immunoinflammatory effects on murine models of infection and malnutrition. The effects of apoE in undernourished and *Cryptosporidium parvum*-infected mice have not been investigated. In order to study the role of apoE in a model of *C. parvum* infection, we used the following C57BL6J mouse genetic strains: APOE-deficient, wild-type controls, and APOE targeted replacement (TR) mice expressing human APOE genes (E3/3; E4/4). Experimental mice were orally infected with 10^7^-unexcysted-*C. parvum* oocysts between post-natal days 34–35 followed by malnutrition induced with a low-protein diet. Mice were euthanized seven days after *C. parvum*-challenge to investigate ileal morphology, cytokines, and cationic arginine transporter (CAT-1), arginase 1, Toll-like receptor 9 (TLR9), and inducible nitric oxide synthase (iNOS) expression. In addition, we analyzed stool oocyst shedding by qRT-PCR and serum lipids. APOE4/4-TR mice had better weight gains after infection plus malnutrition compared with APOE3/3-TR and wild-type mice. APOE4/4-TR and APOE knockout mice had lower oocyst shedding, however the latter exhibited with villus blunting and higher ileal pro-inflammatory cytokines and iNOS transcripts. APOE4/4-TR mice had increased ileal CAT-1, arginase-1, and TLR9 transcripts relative to APOE knockout. Although with anti-parasitic effects, APOE deficiency exacerbates intestinal inflammatory responses and mucosal damage in undernourished and *C. parvum*-infected mice. In addition, the human APOE4 gene was found to be protective against the compounded insult of *Cryptosporidium* infection plus malnutrition, thus extending our previous findings of the protection against diarrhea in APOE4 children. Altogether our findings suggest that apoE plays a key role in the intestinal restitution and immunoinflammatory responses with *Cryptosporidium* infection and malnutrition.

## Introduction

Cryptosporidiosis is a water-borne disease associated with the majority of parasitic protozoan outbreaks recently reported worldwide [1]. Immunocompromised hosts may fail to assemble efficient immune-inflammatory responses against *Cryptosporidium* infections (mainly Th-1-mediated cytokines), leading to intestinal barrier disruption and undernutrition [2].

Malnourished and immune-suppressed children are a particular risk group for *Cryptosporidium* infections, since they are more susceptible to acquiring the infection, more afflicted with a lasting morbidity, and at greater risk of disseminating the infection further, therefore amplifying its spread [3].

A reverberating vicious cycle of infection and malnutrition in young children may cause profound long-term deficits in physical and cognitive development, even without overt diarrhea, which can be irreversible [4]. The predisposition and adverse outcomes from repeated or prolonged exposure to *C. parvum* and other enteric infections may additionally have a strong genetic component in terms of host-parasite and epigenetic interactions [5], [6], which could affect the efficacy of nutritional interventions early in life in children at risk in endemic areas [7] and may have long-term consequences. Indeed, we have found that APOE4, the gene associated with increased susceptibility to Alzheimer's disease, is actually protective against early childhood diarrhea and its associated cognitive impairment [8].

Apolipoprotein E (APOE = gene, apoE = protein) is a critical targeting protein in lipid homeostasis where excess cholesterol from somatic cells helps to form apoE-containing lipoprotein particles that are directed to apoE-receptors in the liver, where the particles are internalized and metabolized [9]. The human APOE gene has 3 alleles at its locus on chromosome 19: APOE2, APOE3, the most frequent allele, and APOE4. APOE4 is often associated with late onset Alzheimer's disease [10], poor recovery after neurological injury [11], and associated with increased oxidative stress [12].

ApoE-deficient mice have been shown to have impaired innate immune defenses in models of *Listeria monocytogenes* [13] and *Klebsiella pneumonia* [14] infections. In addition, in a model of maternal-offspring separation, undernourished APOE knockout mice failed to thrive after being re-fed, showing intestinal mucosal atrophy and poor IGF-1 response [15]. Therefore, we examined the effect of the apolipoprotein-E isoforms in a murine model of *C. parvum* infection plus undernutrition, which we have recently validated [16]. The responses of human APOE targeted replacement and APOE knockout mice to *C. parvum* plus undernutrition were measured to determine whether we could confirm and extend our field studies showing the role of apolipoprotein E in the effects of childhood malnutrition and intestinal infection induced by *C. parvum*. This specific infection/undernutrition/APOE genotype mouse model has permitted us to examine whether pro-inflammatory states associated with APOE transgenic mice [12] would be beneficial against cryptosporidial infections.

## Materials and Methods

### Malnutrition protocol

Thirty-day-old C57BL/6J (APOE^−/−^, APOE^+/+^, APOE3/3 and APOE4/4 targeted replacement male mice) were purchased from Taconic (Albany, New York). Mice were acclimated, body weight matched, and assigned to the experimental groups. Mice assigned to the undernourished groups received an isocaloric diet with 2% of protein (low-protein diet) (Harland Labs, Dublin, VA). Undernourished mice stayed under a low-protein diet for 7 days before *C. parvum* oocyst challenge and continued their diet until the end of the experiment. Nourished control groups received standard chow diet (20% of protein). Weights were monitored daily.

Experimental mice were sacrificed in CO_2_ chambers seven days post-infection (mean age of 42 days old). Euthanasia was assured by cervical dislocation. After opening the abdominal cavity, approximately 1 cm-long ileum segment (proximal to the ileocecal valve) was removed and fixed in 4% paraformaldehyde. Another 2 cm-segment, proximal to the first segment, was removed and milked free of stool for quantitative real-time PCR for cryptosporidial DNA analyses (tissues were frozen and stored in −20°C until assay). A final 1-cm ileal segment was harvested and prepared for real time-PCR and cytokine assays, as described below (tissues were frozen immediately in liquid nitrogen and stored in −80°C until further assays). The protocol described herein was approved and is in accordance with the Institutional Animal Care and Use Committee policies of the University of Virgínia.

### Preparation and administration of inoculum

*C. parvum* unexcysted oocysts were obtained from experimentally infected calves (Iowa isolate; Waterborne, Inc., New Orleans, Louisiana). *C. parvum* oocysts were stored in phosphate-buffered saline (PBS) at 4**°**C and used within 8 weeks of their receipt. The tube with oocysts was gently vortexed and incubated at room temperature for 10 min before use. Each infected mouse received 100 µl of PBS plus freshly prepared unexcysted *C. parvum* oocysts in a recently vortexed solution (10^7^ oocysts per mouse) by oral gavage directly into the stomach. Control mice received 100 µl of PBS by oral gavage at the same time.

### Stool collection and DNA extraction

Stools were collected in pre-weighed tubes daily after gentle abdominal stroking or milked free from the ileum after euthanasia for all groups and stored at −20**°**C until DNA extraction. The DNA was extracted from the frozen stool samples using Qiagen QIAamp DNA Stool Kit (Qiagen, Inc., Germantown, Maryland) with some modifications. First, 400 µl of ASL buffer was added to each sample, vortexed at 1,500 rpm overnight for complete homogenization. Samples were incubated at 82.5**°**C for 5 min and then vortexed for 1 min at full speed. The supernatant was pipetted into a new tube containing 30 µl of proteinase K to which 400 µl of AL buffer was added and incubated at 70°C for 10 min with 400 µl of absolute ethanol and mixed, according to manufacturer's instructions. Finally, DNA was eluted in 200 µl Elution Buffer and stored at −20°C.

### Morphological analyses

Ileal villus heights and crypt depths (n = 4 for each group) were measured using hematoxylin and eosin-stained slides on a light microscope (BH-2, Olympus, Tokyo, Japan), equipped with a high-resolution digital camera that was connected to a computer with an image capture program. Villus height was measured from the baseline to the villus apex. The crypt depth was measured from the baseline to the crypt bottom. All morphometric measurements were done blindly using NIH Image J 1.44 S (National Institutes of Health, Bethesda, MD) after proper calibration.

### C. parvum stool detection by quantitative Polymerase Chain Reaction

Extracted DNA (5 µl) was added to a master mix (20 µl) to give a total reaction volume of 25 µl per sample. The master mix was prepared by mixing 12.5 µl of Bio-Rad iQ SYBR Green Supermix (Bio-Rad Laboratories, Hercules, California), 5.5 µl of DEPC-treated nuclease free sterile water (Fisher Scientific, Pittsburgh, Pennsylvania), and 1.0 µl (6.2 mM) each of both forward and reverse primers (Invitrogen, Carlsbad, California). The primers target the 18 s rRNA gene of the parasite as shown in **Table 1** (GenBank no. AF164102). The reaction was performed in a Bio-Rad iCycler iQ multicolor PCR Detection System using iCycler software (version 3.0). Amplification consisted of 15 min at 95°C followed by 40 cycles of 15 sec at 95°C, 15 sec at 52°C, and 20 sec at 72°C, followed by 0.5-degree increments for 10 sec starting at 75°C and ending with 95°C for the Melt Curve. Fluorescence was measured during the annealing step of each cycle. Ct values of each run were compared to standards with known amounts of *C. parvum* DNA and log transformed into number of organisms per mg of stool sample.

**Table 1:** Primers used in the study.

Primers	Sequence (5′-3′)
**Beta-actin**	AATTTCTGAATGGCCCAGGT TTTGTGTAAGGTAAGGTGTGC
**Arginase -1**	TCTGCCAAAGACATCGTGTA GGTAGCTGAAGGTCTCTTCC
**CAT – 1**	CACTGCTGATCTGTGTACCT GTGGGGACATAAGATGCTCA
**RNA 18 s ** ***C. parvum***	CTGCGAATGGCTCATTATAACA AGGCCAATACCCTACCGTCT
**iNOS**	TCCTGGACATTACGACCCCT AGGCCTCCAATCTCTGCCTA
**TLR9**	TGGTGTTGAAGGACAGTTCTCTC CACTCGGAGGTTTCCCAGC

### Inflammatory Cytokine Beads Assay (Luminex)

Ileal specimens were stored at −80°C until required for assay. The harvested tissue was homogenized and processed with HEPES buffer (Invitrogen, Carlsbad, CA, USA) (pH = 7.4). Briefly, microtiter plates were coated overnight at 4°C with antibodies against murine TNF-α, IL-1β and IL-10 (2 µg/ml). After blocking the plates, the samples and standards at various dilutions were added in duplicate and incubated at 4°C for 2 h. The plates were washed three times with buffer. After washing the plates, biotinylated sheep polyclonal anti-TNF-α or anti-IL-1β or anti-IL-10 (diluted 1∶1000 with assay buffer 1% BSA), was added to the wells. After further incubation at room temperature for 1 h, the plates were washed and 50 µl of avidin-HRP diluted 1∶5000 was added. The color reagent phenylenediamine (OPD; 50 µl) was added 15 min later and the plates were incubated in the dark at 37°C for 15–20 min. The enzyme reaction was stopped with 2 N H_2_SO_4_ and absorbance was measured at 490 nm. Values were expressed as picograms/mililiter (pg/ml).

### Serum lipid measurements

The samples were collected at the end of experiment (day 7 after infection). Blood was drawn in anesthetized animals through transcardiac puncture using a 1 ml-syringe. The blood was transferred to a PCR tube and coagulated at room temperature. Tubes were then centrifuged (3.000 rpm) for 10 minutes at 4°C. The serum was stored at −80°C until lipid analyses were performed according to the method of Roeschlau and colleagues [17].

### CAT-1 and Arginase 1 qRT-PCR analyses (quantitative reverse transcriptase-polymerase chain reaction)

Total cellular RNA was obtained from each intestinal tissue using the RNeasy kits (Qiagen), and cDNA was synthesized from 1 µ, and cDNA wiScript (Biorad). For quantitative PCR analyses of cytokine mRNA abundance, cDNA was diluted 1∶8; 4 µL of this dilution were used for each PCR reaction. Reagents from the BioRad real-time PCR kit containing Sybr Green were used for quantitative PCR reactions. Primer sequences used in the experiment are shown in the **Table 1**.

The PCR conditions were: 95°C 10 min, 95°C 3 min, followed by 40 cycles of 95°C 30 s, 58°C 30 s, and 72°C 30 s, followed by a melt curve analysis. Data were analyzed and are presented based upon the relative expression method [16]. The formula used for calculation was: Relative expression = 2^−(**S**Δ**CT- C**Δ**CT)**^, where Δ**CT** is the difference in threshold cycle between the gene of interest (i.e., Arginase-1) and the housekeeping gene (β-actin)**S** = *C. parvum* challenged mice and **C** = uninfected mice.

### Statistical analysis

Analyses of weight loss/increase were expressed as percent change in baseline body weight. The analyses of stool shedding (number of parasites per mg of stool) were performed using Graph Pad Prism version 5.0 software (Media Cybernetics, CA). Statistical analyses were performed using ANOVA with Bonferroni post-hoc correction or unpaired Student *T* test when appropriate. A *P* value<0.05 was considered significant. Data are presented as mean ± standard error, with the exception of serum lipid levels which are presented as mean ± standard deviation.

## Results

### APOE 4/4 TR mice lost less weight when challenged by under nutrition and cryptosporidiosis

**Figure 1:**
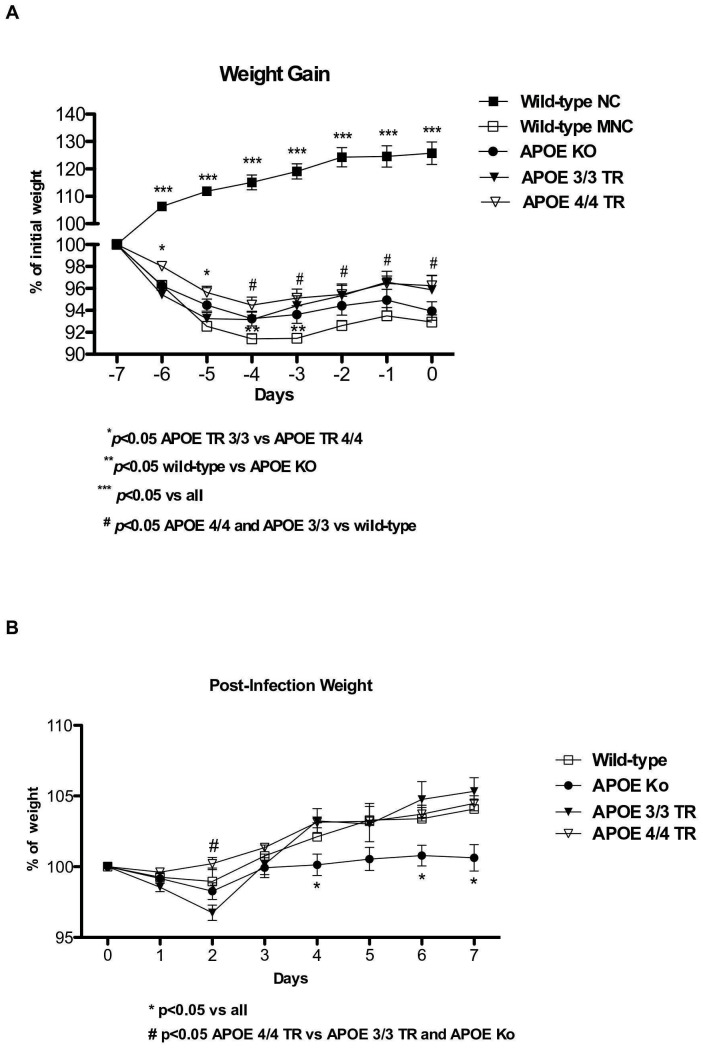
A. Body weight gain (% initial weight) from experimental uninfected and undernourished groups under a low protein diet. APOE 4/4 targeted replacement (APOE 4/4 TR) mice (n = 17) showed 643 a better growth response in comparison with APOE 3/3 targeted replacement (APOE 3/3 TR) mice (n = 8). **B.** Body weight gain (% initial infection weight) from experimental mice challenged by a compounded malnutrition and *Cryptosporidium parvum* insult. Undernourished mice were orally inoculated with 10^7^- unexcysted oocysts diluted in 100 µl of PBS. APOE deficient mice show impaired growth following *Cryptosporidium parvum* infection as compared to the other groups. Results are shown as mean ±SEM.

The inability to reach an expected growth rate has been proposed as a biological marker of under nutrition and/or enteric infections in animal models [15], [18]. All experimental groups under the low protein diet showed significant short-term weight loss, as compared with the wild-type nourished controls, however APOE 4/4 targeted replacement mice had less weight loss with under nutrition compared with APOE 3/3 and wild-type undernourished mice. APOE knockout mice showed a slightly better weight adaptation than wild-type controls under the low-protein diet (**Fig. 1A**).

After *Cryptosporidium parvum* infection, undernourished APOE 4/4 TR mice had less weight decrements, at day 2 post-inoculum, when the parasite load was heavier (see below) when compared with undernourished APOE knockout and APOE 3/3 TR. APOE3/3 TR mice had the greatest weight loss on day 2. In addition, undernourished infected APOE knockout mice showed significant weight impairments after the 4th day of *C. parvum* challenge and showed poor growth catch-up after infection (**Fig. 1B**).

### APOE 4/4 TR mice have significantly lower fecal C. parvum oocyst shedding

**Figure 2:**
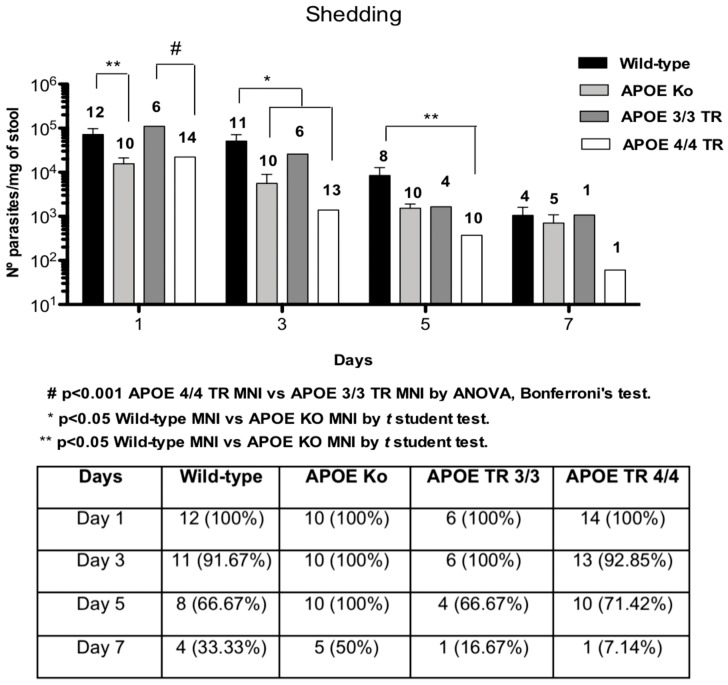
Fecal shedding of parasites in weaned undernourished C57BL/6 mice orally inoculated with 10^7^-unexcysted *Cryptosporidium parvum* oocysts per mouse (given in100 µL of PBS) on day 7 after the onset of the low protein diet. Results are shown in a log scale a mean±SEM. *Cryptosporidium parvum* stool oocyst shedding was determined by qRT PCR. Data were expressed as number of parasites per miligram of stool and percentage and number of infected mice with measurable oocyst shedding per day after *Cryptosporidium parvum* challenge. N above the bars means the number of mice still showing oocyst shedding.

To better evaluate *C. parvum* infection in undernourished experimental mice, we assessed the quantity of *C. parvum* oocyst shedding in stools using quantitative polymerase chain reaction. From day 3 after infection onward, the APOE 4/4 TR mice showed an accelerated pace of oocyst reduction. After one week post-infection, only one (1 out of 14, 7.4%) experimentally infected –APOE 4/4 TR mouse showed *C. parvum* shedding in stool (**Fig. 2**). In addition, the APOE knockout mice had slightly, but significantly lower oocyst shedding per milligram of stool (p<0.05) than APOE3/3 TR mice at days 1 and 3 post- infection, when the oocyst burden in stool was higher.

### APOE 4/4 TR have improved intestinal villi when challenged by under nutrition and cryptosporidiosis

**Figure 3:**
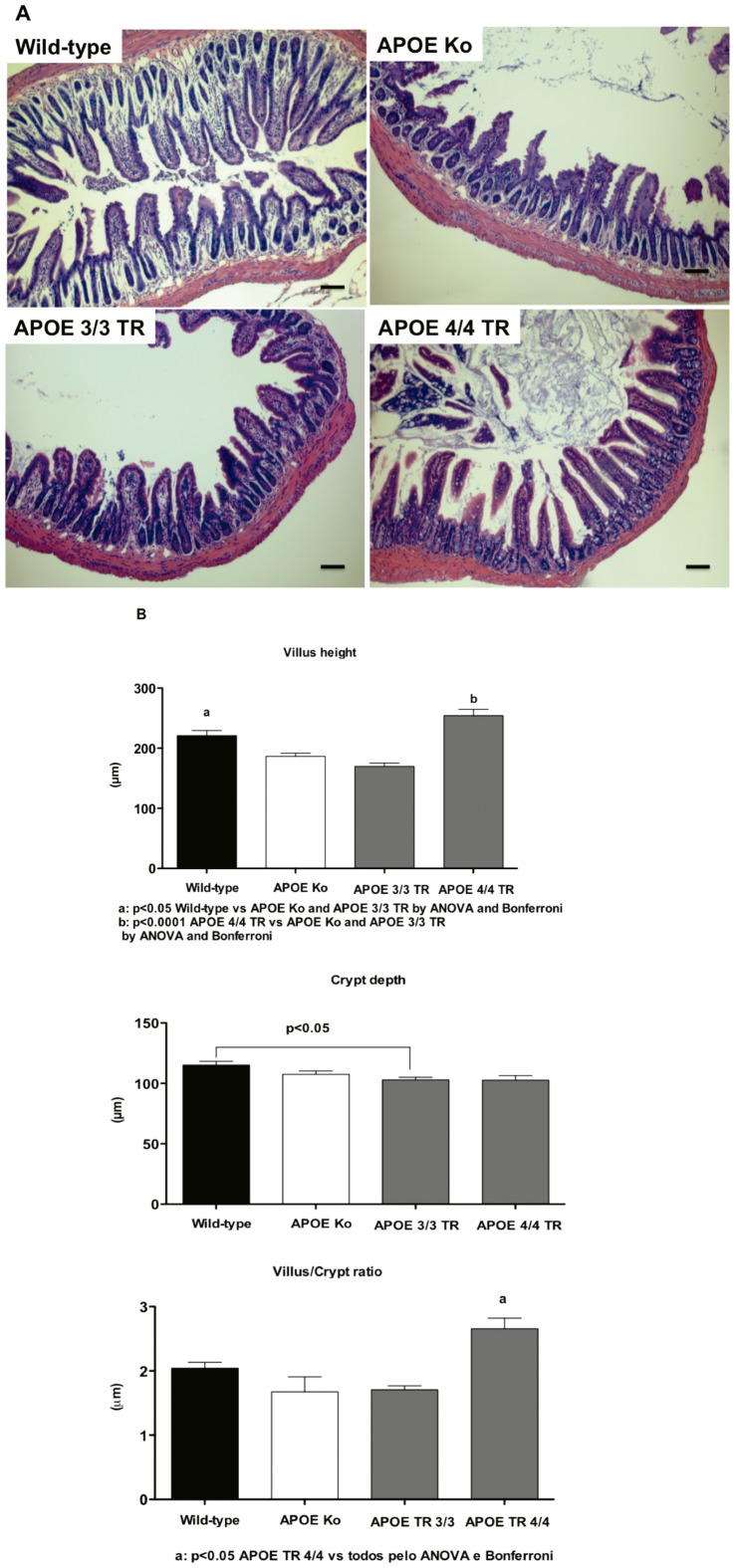
A. Representative ileal histology from orally *Cryptosporidium parvum* infected mice. Wild-type, APOE knock-out, and APOE targeted replacement mice (APOE 3/3 TR and APOE 4/4 TR) were fed with a low protein diet during 7 days then infected with 10^7^-unexcysted *Cryptosporidium parvum* oocysts and euthanized seven days after infection. H&E ×400. Scale bar 10 µM. **B**. Ileal villus height; **C**. crypt depth, and **D**. villus-crypt ratio from wild-type, APOE knock-out, and APOE targeted replacement mice (APOE 3/3 TR and APOE 4/4 TR). Morphometrics was done from hematoxylin and eosin stained-sections in at least four animals per group at low magnification. Data are presented as mean±SEM. Comparisons were performed by Students unpaired *T* test. Villi and crypts were measured only when their full longitudinal axis was found.

Undernourished uninfected mice showed ileal villus shortening and crypt derangement, with almost complete absence of mitoses. Cryptosporidial infections were associated with crypt hyperplasia, villus blunting, and inflammation in the lamina propria and submucosa as opposed to nourished controls (data not shown). Infected undernourished APOE knockout mice showed reduced villus height and more scattered villi as compared to wild-type and APOE4/4 TR. APOE 3/3 TR mice presented slightly reduced crypt depth as compared to wild-type mice (**Fig. 3A, B and C**). Furthermore, APOE4/4 TR showed better villus height as compared to APOE 3/3 TR and APOE knockout mice following infection and under nutrition (p<0.05) (**Fig. 3A and B**). In addition infected APOE 4/4 TR showed better villus/crypt ratio as opposed to all other challenged groups (**Fig. 3D**).

### APOE 4/4 TR mice have lower serum triglyceride levels when challenged by under nutrition and cryptosporidiosis

In order to find whether the host response to infections involves changes in lipid levels, we measured serum lipid fractions. Following *C. parvum* infection in undernourished mice, we found significant increases (8 fold higher) in serum total cholesterol and LDL-cholesterol in APOE knockout mice than in C57BL/6J controls.

The APOE 4/4 TR mice had a trend of increased serum HDL cholesterol levels when compared with the other groups. No significant differences were found between APOE 3/3 and APOE 4/4 TR mice. The APOE knockout mice had increased serum triglyceride levels in comparison to APOE 4/4 TR mice. In addition, albeit not significant, APOE 3/3 TR mice showed higher serum triglyceride levels (**Table 2**).

**Table 2:** Lipid profile of experimental undernourished mice following *Cryposporidium parvum* infection (mice orally infected with 10^7^ unexcysted oocysts).

Analytes (mg/dL)	Wild-type (n = 5)	ApoE Ko (n = 5)	ApoE TR 4/4 (n = 5)	ApoE TR 3/3 (n = 4)
**Total Cholesterol**	60.8±18.5	615.8±69.6*	112±16.9	96.75±14.0
** HDL**	52±9.3	50±5.7	58±6.2**	47±7.8
** LDL**	29.80±6.5	576±65.8***	45.8±10.4	38.50±7.0
**Triglycerides**	53.20±17	59±4.0#	48.6±8.0	68.5±24.0

*p<0.001 ApoE Ko vs all;

** p = 0.05 ApoE TR 4/4 vs ApoE TR 3/3;

*** p<0.001 ApoE Ko vs all;

# p<0.05 ApoE Ko vs ApoE TR 4/4 by Student *t* Test.

### Undernourished APOE knockout mice have increased ileal pro-inflammatory cytokines with cryptosporidial infection

Ileal cytokine analyses revealed significantly higher IL-1β and IFN-γ levels in APOE knockout mice following *C. parvum* infection and undernutrition as compared with respective wild-type controls. In general, there were heavier inflammatory cytokine responses associated with apoE deficiency. Furthermore, both APOE TR undernourished and infected mice showed lower ileal levels of IL-1β than APOE knockout and wild-type challenged mice.

**Figure 4:**
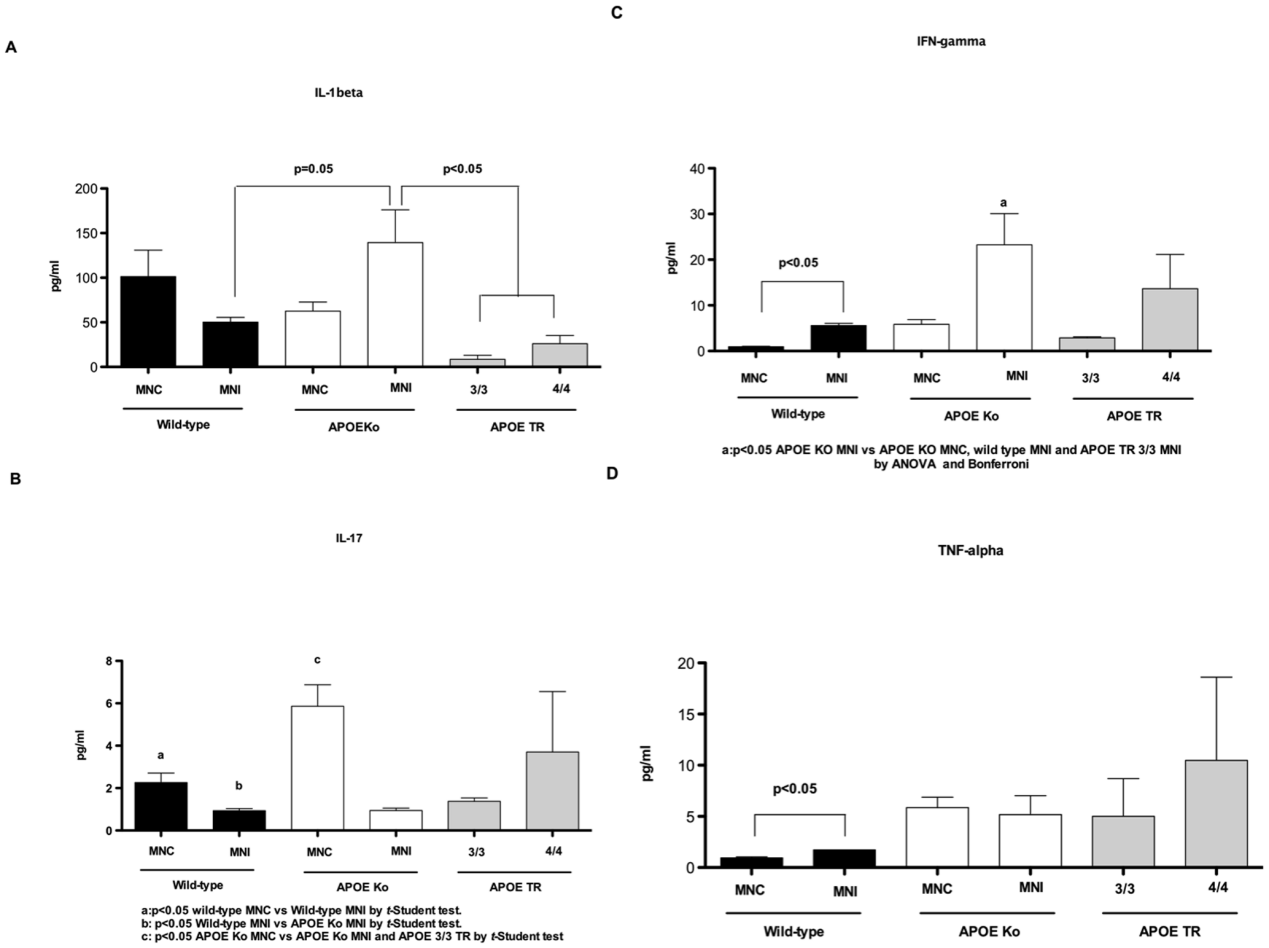
Luminex assays from experimental mice for the following ileal pro-inflammatory cytokines: (A) Interleukin 1-β; (B) Interleukin-17; (C) Interferon-gamma; and (D) Tumor necrosis factor alpha. Experimental mice were challenged by a compounded malnutrition and *Cryptosporidium parvum* insult and samples were harvest on day 7 post-*C. parvum inoculum*. Wild-type, APOE knock-out, and APOE targeted replacement mice (APOE 3/3 TR and APOE 4/4 TR) were orally inoculated with 10^7^-unexcysted oocysts diluted in 100 µl of PBS. Groups have at least 4 per groups and the results are shown as mean ±SEM and are expressed in pg/ml. MNC = uninfected undernouorished control group. MNI = undernourished infected group.

IFN-γ and TNF-α are key cytokines related to *C. parvum* eradication. They were both increased in wild-type undernourished infected mice in comparison with uninfected controls. On the other hand, IL-17 levels were lower in APOE knockout mice after infection and under-nutrition as compared with the APOE knockout uninfected controls and with undernourished infected wild-types. No significant differences were seen between APOE3/3 and APOE4/4 TR mice in all cytokines measured (**Fig. 4**).

### APOE 4/4 TR mice showed increased CAT-1, arginase 1, and TLR-9 mRNA when challenged by under nutrition and cryptosporidiosis

**Figure 5:**
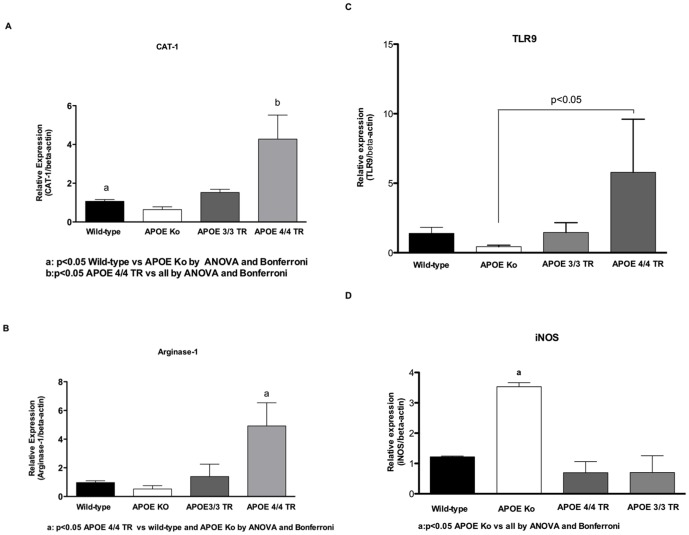
Quantitative real-time PCR assays from experimental mice for the following ileal mRNA transcripts: (A) cationic amino acid transporter (CAT-1); (B) arginase 1; (C) Toll-like receptor 9 (TLR9); and (D) Inducible nitric oxide synthase (iNOS). Experimental mice were challenged by a compounded malnutrition and *Cryptosporidium parvum* insult and samples were harvested on day 7 post-*C. parvum inoculum*. Wild-type, APOE knockout, and APOE targeted replacement mice (APOE 3/3 TR and APOE 4/4 TR) were orally inoculated with 10^7^- unexcysted oocysts diluted in 100 µl of PBS. Groups have at least 4 per groups and the results are shown as mean ±SEM and expressed after β-actin normalization.

In order to assess key mechanisms involved in host-parasite interactions and tissue repair, we addressed the cationic amino acid transporter (CAT-1), arginase-1, and Toll-like receptor 9. We found increased ileal expression of CAT-1 mRNA transcripts in the ileum from undernourished plus infected APOE 4/4 TR mice in comparison to wild-type and APOE-deficient mice on day 7 post-infection. In addition, APOE knockout mice had significantly reduced ileal CAT-1 mRNA expression as compared to wild-type mice (p<0.05). Similar changes were also seen with arginase 1, where the highest ileal levels were observed in APOE4/4 TR mice (**Fig. 5 A and B**). In addition we found an increased ileal mRNA levels of TLR9 (Toll-like receptor 9) in APOE 4/4 TR group in comparison to APOE knockout mice (p<0.05). Increased ileal levels of inducible nitric oxide synthase (iNOS) mRNA were found in APOE knockout mice in comparison to all groups (p<0.05) (**Fig. 5C and D**).

## Discussion

Children in developing areas are particularly susceptible to malnutrition associated with *Cryptosporidium parvum* infections, resulting in debilitating diarrhea as well as growth shortfalls [19]. Since nutritional states and the APOE genetic profile may directly affect the host response against infection [20], [21] and therefore influence the risk of acquiring and spreading *Cryptosporidia*, we evaluated this infection in different undernourished C57BL6J genetically-engineered mice, including wild-type, APOE-deficient and APOE target-replacement mice.

In this study, we found that APOE-deficient mice had significantly less *C. parvum* oocyst shedding measured by quantitative PCR in stools on the first and third days post-infection, when the infection peaks. In agreement with our findings, greater mucosal immune responses against orally-inoculated pathogens, via Th-1 responses, have been shown in APOE knockout mice [22]. In addition, our data suggest that these parasite-killing effects are independent of any increases in IL-17, which was actually found to be low in the ileal tissue of these animals. The Th-17-mediated pathway has been reported to coordinate the immune response against extracellular bacteria [23], suggesting that APOE deficient mice may be poorly adapted against the potential intestinal bacterial translocation due to *C. parvum*-induced intestinal barrier leakage.

Emulsion associated and free-apoE have been shown to bind to LPS (by its exposed hydrophilic domain involving arginine residues) and to redirect LPS from Kupffer cells to liver parenchymal cells, altering lipid metabolism and improving LPS clearance via bile from circulation and reducing LPS-induced lethality [24], [25].

Although exhibiting more parasite elimination, undernourished APOE-deficient mice had greater inflammatory cytokine responses and mucosal atrophy in the ileal tissue one week-post inoculum, accompanied by greater weight deficits following 7 days of infection. This can be explained by a constitutive pro-inflammatory state that has been shown with ApoE deficiency in association with hypercholesterolemia [12]. Hypercholesterolemia found in the *C. parvum*-infected APOE knockout mice, even sustained following under nutrition, as shown in our study, may reflect impaired intestinal cholesterol delivery to the liver for metabolism. This effect may be associated with increased bile acid transport in the terminal ileum and diminished fecal bile acid loss, and higher serum and intestinal LDL-cholesterol levels [26].

The transient, slightly better gain weight seen in uninfected APOE knockout mice on the low-protein diet, as compared with wild-type controls, may reflect more available cholesterol, regardless of ApoE/lipoprotein delivery, used as an energy source for growth. Prentice and colleagues have shown that energy-enriched diets are critical for catch-up growth following acute enteric infections [27]. In our earlier studies, we found that neonatal undernourished APOE knockout mice failed to thrive after being re-fed [15]. These seemingly slightly contradictory findings may be explained by the low body-fat mass in the neonatal period that would render these mice with insufficient cholesterol to support rapid growth or the high cholesterol phenotype in APOE knockout mice may occur later after weaning.

Overproduction of TNF-α, IL-1β, and decreased IL-10 ileal levels seen in undernourished and infected ApoE knockout mice could be a consequence of the intestinal barrier disruption and increased luminal LPS-trafficking to the lamina propria. Infections and increased LPS intestinal transit (or cytokines activated by it), even in low amounts, could lead to a hypertriglyceridemic state altering liver lipid metabolism [28]. Endotoxin stimulates arginine transport via TNF-α signaling [29], thus impaired intestinal arginine uptake seen with ApoE deficiency (but improved in APOE4 targeted replacement mice) could contribute to poor bacterial removal and increased mucosal inflammation.

Lipoproteins and lipids present in the serum may contribute to the host innate immunity against pathogens [30]. Studies using APOE deficient mice confirmed the role of APOE in host susceptibility to endotoxemia and *Klebsiella pneumoniae* infection [14], while transgenic mice expressing human APOE3 and APOE4 genes revealed an isoform-specific effect of APOE on the proinflammatory response to lipopolysaccharide [31].

Toll-like receptors are key mediators of the innate immune system against *C. parvum* infections [32]. In our previous study, we have shown that undernourished C57BL6J mice had higher ileal mRNA transcripts for TLR2 and TLR4, but not TLR9, which was diminished by *C. parvum* infection [16]. In the current study, we found that APOE4 mice had increased ileal TLR9 transcripts compared with APOE knockout mice. TLR9-immune mediated responses have been found important to control C. *parvum* infection in a neonatal mouse model [33].

APOE4 may also contribute to elimination of *C. parvum* infection with an inflammatory response that is more regulated compared to the uncontrolled cytokine production noted in ApoE deficient mice. ApoE also enhances microbial lipid antigen presentation (which can be found with increased bacterial intestinal translocation) to antigen-presenting cells via the low-density lipoprotein receptor (LDLR) which could culminate in natural killer T (NKT) cell activation and cytokine secretion [34], [35], an effect that could attenuate *C. parvum* infection.

Finally, APOE4 has been shown to up-regulate the L-arginine selective cationic protein transporter (CAT-1), part of the amino acid-polyamine-organocation (APC) superfamily, which has been associated with increased L-arginine uptake by neuroglia cells. Our data show that CAT-1 is up-regulated in the ileum of undernourished *C. parvum* infected-APOE4 targeted replacement mice compared to APOE3 controls. Increased uptake of L-arginine preferentially by enterocytes may enhance the intestinal barrier function, mucosal blood flow, and the immune system in addition to mucosal repair (cell proliferation and migration) following injury, by increasing polyamine synthesis and constitutive nitric oxide synthase (cNOS) activity [36]. Indeed, we have observed that arginine can enhance the killing of cryptosporidial parasites in our murine model, via both iNOS and arginase pathways [37]. In addition, protozoa could utilize arginine and therefore reducing the NO production by the host and weakening the innate immune defenses against infection [37].

Arginase-1 is an enzyme that catalyzes arginine's conversion into ornithine, which may then enter into the polyamine pathway [38]. Taken with our previous findings and those Colton et al. see below and reference [46], these findings with ApoE 4/4 targeted replacement transgenic mice suggest that increased uptake of arginine through CAT-1 activity and the arginine shift to the arginase pathway for improving mucosal restitution following *C.parvum*-induced mucosa injury in undernourished mice.

Recently, we have demonstrated that L-arginine supplementation improved mucosal recovery following *C. parvum* infection plus undernutrition and that ileal arginase 1 was a marker of infection severity [37]. In contrast with that finding, APOE4 targeted replacement mice up-regulated arginase 1 which may indicate that mucosal recovery is still underway. More studies are warranted to evaluate the role of arginase 1 during *C. parvum* infection and mucosal recovery.

APOE4 targeted gene replacement and APOE knockout mice have been found with increased pro-inflammatory states [39], an effect that is potentially undesirable for neurodegenerative diseases, but beneficial during exposure to gut-infectious pathogens early in life [40]. The beneficial effect of apoE4 in boosting host innate immunity against infectious agents is a potential explanation for the prevalence of the APOE4 in our genetic pool [41], and recognized as a thrifty allele [42]. This gene is considered important to enhance energy usage and storage and to foster host defenses at times of food scarcity and when repeated bouts of infection outbreaks occur and energy usage for catch-up growth and fit immunity are critical requirements for immediate survival [43].

Interestingly, data from the Tsiname population in lowland Bolivia (an indigenous forager-farmer population, with high infectious morbidity and shortened life expectancy) when bearing APOE4 show lower C-reactive protein levels, suggesting low rates of environmental-related infections [44]. In our study, targeted transgenic mice expressing human apoE4 protein have an advantage in eliminating *C. parvum* oocysts after being exposed to a high load of parasitic inoculum supporting the concept of antagonistic pleiotropy for the APOE4 gene [45].

Altogether, our findings support that APOE4 is protective during *C. parvum* infections early in life, improving parasite elimination with enhanced intestinal mucosal recovery. These effects may be caused by boosting innate immunity but without the overt inflammation seen with apoE deficiency and improving arginine uptake and arginase 1-driven mucosal restitution.

The APOE4 allele has been associated with increased nitric oxide production in platelet cells and in macrophages also is associated with increase expression of CAT-1 (cationic amino acid transporter), and with increased arginine uptake in brain microglia [46].

Our mouse model data support the beneficial effect of the APOE4 allele seen in cohort shantytown children afflicted with malnutrition and enteric infections who harbor this allele [20]. Although the orchestrated epithelial restitution and immune-inflammatory responses against malnutrition and enteric infections constitute a multi-factorial gene-to-gene interplay, the APOE gene appears to enable better intestinal adaptation against *Cryptosporidium parvum*.

One limitation of this study is the lack of the undernourished uninfected controls for comparisons of baseline levels for the APOE TR 4/4 and TR3/3 mice. In addition, we cannot rule out that the level of *Cryptostoridium* excystation after oral inoculation may have varied in the different genetic mouse strains used, however, after 6 h of *C. parvum* challenge in a model of human HCT-8 epithelial monolayers a considerable excystation of the oocysts is already seen [47].

In summary, our overall findings suggest that ApoE is one of the key players modulating the intestinal architecture and immune and inflammatory responses following malnutrition and *C. parvum* infection.
